# Iodine Injections into the Peritoneum in Ascites

**Published:** 1852-05

**Authors:** 


From the London Lancet.

*Iodine Injections into the Peritoneum in Ascites.*

WE have already spoken at some length, but very guardedly, of M. Boinet's method of treating ascites; injections of iodide of potassium and tincture of iodine into the cavity of the peritoneum, seem, *prima facie*, measures of a very hazardous kind; but we now find that M. Boinet has published, in the *Gazette Medicale de Paris* (No. 47, 1851,) an account of thirteen cases, wherein he has used such injections with perfect success as to eleven of these; the two remaining cases, though unsuccessful, not presenting any further unpleasant symptoms than the persistence of the ascites. Though the febrile reaction never ran high, the injections were in general followed by tension and some heat of the abdomen, a little tympanitis, and tenderness on pressure. In two or three cases there were slight symptoms of peritonitis, and in one case, intense inflammation of the peritoneum. The fluid was generally left four or five minutes, and it has even happened that from difficulty of evacuating it, one fourth, one half, or even the whole, was left without producing unpleasant symptoms. Of course, the treatment will only apply in cases of idiopathic affection of the peritoneum, unconnected with organic disease. The injection should be composed as follows: water, seven ounces; tincture of iodine, one ounce; iodide of potassium, one drachm. The injection must never contain more than a sixth or seventh part of iodine.

